# Clinical and kinematic characterization of parkinsonian soft signs in essential tremor

**DOI:** 10.1007/s00702-024-02784-0

**Published:** 2024-05-14

**Authors:** Luca Angelini, Giulia Paparella, Antonio Cannavacciuolo, Davide Costa, Daniele Birreci, Martina De Riggi, Massimiliano Passaretti, Donato Colella, Andrea Guerra, Alfredo Berardelli, Matteo Bologna

**Affiliations:** 1 IRCCS Neuromed, Via Atinense, 18, Pozzilli (IS), 86077 Italy; 2 Department of Human Neurosciences, Sapienza University of Rome, Viale dell’Università, 30, Rome, 00185 Italy; 3 Parkinson and Movement Disorders Unit, Study Center on Neurodegeneration (CESNE), Department of Neuroscience, University of Padua, Padua, Italy; 4 Padova Neuroscience Center (PNC), University of Padua, Padua, Italy

**Keywords:** Essential tremor, Rest tremor, Bradykinesia, Neurophysiology

## Abstract

**Background:**

Subtle parkinsonian signs, i.e., rest tremor and bradykinesia, are considered soft signs for defining essential tremor (ET) plus.

**Objectives:**

Our study aimed to further characterize subtle parkinsonian signs in a relatively large sample of ET patients from a clinical and neurophysiological perspective.

**Methods:**

We employed clinical scales and kinematic techniques to assess a sample of 82 ET patients. Eighty healthy controls matched for gender and age were also included. The primary focus of our study was to conduct a comparative analysis of ET patients (without any soft signs) and ET-plus patients with rest tremor and/or bradykinesia. Additionally, we investigated the asymmetry and side concordance of these soft signs.

**Results:**

In ET-plus patients with parkinsonian soft signs (56.10% of the sample), rest tremor was clinically observed in 41.30% of cases, bradykinesia in 30.43%, and rest tremor plus bradykinesia in 28.26%. Patients with rest tremor had more severe and widespread action tremor than other patients. Furthermore, we observed a positive correlation between the amplitude of action and rest tremor. Most ET-plus patients had an asymmetry of rest tremor and bradykinesia. There was no side concordance between these soft signs, as confirmed through both clinical examination and kinematic evaluation.

**Conclusions:**

Rest tremor and bradykinesia are frequently observed in ET and are often asymmetric but not concordant. Our findings provide a better insight into the phenomenology of ET and suggest that the parkinsonian soft signs (rest tremor and bradykinesia) in ET-plus may originate from distinct pathophysiological mechanisms.

## Introduction

Essential tremor (ET) is a common movement disorder characterized by bilateral action tremor of the upper limbs. The ET diagnosis requires no other signs of neurological disorders to be present (Bhatia et al. 2018). Recently, the term ET-plus has been introduced to describe cases of ET characterized by additional mild neurological signs, specifically soft signs, of uncertain significance but not sufficient to warrant alternative diagnoses (Bhatia et al. 2018). In the context of tremor classification, the ET-plus is a topic of debate (Louis et al. 2020; Erro et al. 2022c; Vidailhet 2020). It remains unclear whether the ET-plus represents an advanced stage of ET (Iglesias-Hernandez et al. 2021; Angelini et al. 2023), or a distinct subtype and whether it should prompt consideration of alternative diagnoses (Erro et al. 2022b). Among the soft signs, two main symptoms of parkinsonism, rest tremor and bradykinesia, can be present in ET-plus patients (Bologna et al. 2020; Erro et al. 2022a, b). In particular bradykinesia in ET is characterized by slowness of voluntary movement without sequence effect or rhythm irregularities (Bologna et al. 2020; Paparella et al. 2023). In accordance with a recent position paper published by our group (Bologna et al. 2023), hereafter we will use the term bradykinesia in a literal sense to refer exclusively to movement slowness.

To gain further insights into the significance of rest tremor and bradykinesia in ET-plus, it is worth investigating their relationship with action tremor, their possible asymmetry and side concordance. Indeed, the degree of rest tremor asymmetry in ET has been explored only in a limited number of studies (Cohen et al. 2003; Louis et al. 2011, 2015; Delgado et al. 2022), most of them clinical. Also, no studies have examined the degree of bradykinesia asymmetry in ET. Finally, it is unknown whether there is a side concordance of rest tremor and bradykinesia in ET, which could be relevant for pathophysiological reasoning.

A better comprehension of the ET-plus heterogeneity through an objective neurophysiological evaluation can provide advances in understanding the pathophysiology that underlie this clinical entity. This approach can help to understand whether alterations in a single brain circuit or in different networks underpin the different motor symptoms of ET, and how they interact with each other in determining clinical manifestations.

For this purpose, we conducted a comprehensive clinical and kinematic assessment of rest and action tremor in ET, as well as an analysis of repetitive finger voluntary movements to evaluate bradykinesia. Our objective was to investigate the side distribution and concordance of parkinsonian soft signs, and their relationship with action tremor in a relatively large sample of ET and ET-plus patients.

## Methods

### Participants

Eighty-two right-handed patients diagnosed with ET and ET-plus with rest tremor and/or bradykinesia, were consecutively recruited from the Department of Human Neurosciences outpatient clinic at Sapienza University of Rome. A healthy control group (HC) of 80 subjects matched for gender and age was included for the comparison of finger tapping movement velocity. Healthy subjects were recruited from non-blood related caregivers of patients followed in our movement disorders outpatient clinic. Subjects were required to have no neurological or other clinical conditions with potential impact on motor function, nor were they to be taking medications known to alter motor performance itself. Three neurologists with expertise in movement disorders (LA, GP, MB) were responsible for recruitment; the most recent diagnostic criteria were used (Bhatia et al. 2018). A dopamine transporter (DAT) scan using single-photon emission computed tomography (SPECT) was performed when judged helpful for clinical purposes (in 3 patients for the presence of rest tremor and bradykinesia, in 2 for the presence of clinically evident bradykinesia, and in 5 for the predominance in amplitude of the rest tremor over the action tremor) to rule out a central dopaminergic deficit indicative of parkinsonism, and proved negative in all cases. Regarding parkinsonian soft signs, subjects scoring at least 1 point in the Movement Disorder Society-sponsored revision of the Unified Parkinson’s Disease Rating Scale (MDS-UPDRS) item 3.17 for upper limbs rest tremor (‘slight: < 1 cm in maximal amplitude’) and item 3.4 for bradykinesia considering only movement speed (‘slight slowing’) were included. The presence of mild cognitive impairment (MCI) and impaired tandem gait (ITG) were evaluated in relation to the parkinsonian soft signs without further characterization. Similarly, we did not focus on the description of all other soft signs, including questionable dystonia, because although possibly present in some ET-plus patients they are considered beyond the scope of this paper and in any case with minimal influence on the present data.

The experimental procedures adhered to the principles of the Declaration of Helsinki and were approved by the local ethics committee. All participants provided written informed consent to participate in the study.

### Clinical evaluation

Patients underwent a thorough neurological examination, which included the Fahn-Tolosa-Marin Tremor Rating Scale (FTM-TRS) (Fahn et al. 1988) and the MDS-UPDRS Part III (Goetz et al. 2008). For rest tremor, we clinically ascertained that patient had their upper limbs completely relaxed. Global cognition was also assessed by the Montreal Cognitive Assessment (MoCA) (Nasreddine et al. 2005), and a complete neuropsychological evaluation was executed by an expert neuropsychologist to evaluate the presence of mild cognitive impairment (MCI), with the cut-offs used in previous works (Bologna et al. 2019; Angelini et al. 2023). In particular, the test battery included the evaluation of executive function (Raven’s Progressive Matrices, Stroop test, phonemic fluency, category fluency, Frontal Assessment Battery - FAB), attention (Trail Making Test, versions A and B, Visual Search), memory (Rey Test, Babcock Story Recall Test (BSRT), long-term memory, working memory, visuospatial memory), and visuo-constructional (Rey-Osterrieth Complex Figure test). Raw scores were corrected for age and years of education using normative data available for each test and converted to z-scores. Z-scores ≤ -2 were considered indicative of test impairment. Two impaired executive function tests were required for a patient’s executive function domain to be considered impaired, while only one impaired test was required for all the other domains (Collins et al. 2017; Bologna et al. 2019; Angelini et al. 2023). ITG was considered to be present in subjects who scored at least 1 in item 1 of the Scale for the Assessment and Rating of Ataxia (SARA).

Rest tremor and bradykinesia were considered clinically asymmetric if present in only one side of the body or if there was a difference of at least 1 point between the two sides for the relevant items of the MDS-UPDRS Part III. Similarly, clinical asymmetry in action tremor of the upper limbs was defined by a difference of at least 1 point between the two sides in the posture and/or action/intention subscores of items 5 and 6 of the FTM-TRS.

### Kinematic recordings and analysis

The assessment was conducted as in previous works (Bologna et al. 2019, 2020; Angelini et al. 2023). Postural tremor was measured in two different arm positions in three 45-seconds recordings for each position: arms outstretched in front of the chest (posture 1 – P1) and arms flexed at the elbows in a lateral “wing beating” posture (posture 2 – P2). Three 45-second recordings were performed for rest tremor assessment, with patients seated comfortably on a chair with their arms fully relaxed and placed on a table in front of them. Additionally, three 15-second recordings were taken for each arm to assess kinetic tremor during repetitive arm movements (Bologna et al. 2019, 2020; Angelini et al. 2023). For postural and rest tremors of the upper limbs, the signal was filtered with a bandpass filter at 3–12 Hz. The power spectrum was then obtained for each track using a Welch periodogram with a segment length of two seconds and a Hammer taper. Tremor was considered present in a track if a clear peak with half-power bandwidth narrower than 2 Hz was present (Deuschl et al. 2022). The tremor frequency peak expressed in Hz was selected for each patient, considering the average value of the three axes in the different tracks when differences existed. The tremor amplitude was then determined by measuring the tremor power at the individual frequency peak ± 1 Hz in the three axes of space, and calculating the magnitude of the accelerometer vector with the formula $\sqrt{{x}^{2}+{y}^{2}+{z}^{2 }}$ (Janidarmian et al. 2017) for each track. The average amplitude values of tacks under the same activation condition were considered and expressed in the squared acceleration root mean square (GRMS^2^). For postural tremor, the average values of the two postures were considered. For kinetic tremor, an algorithm was used to calculate the curvature index (CI) as an indirect measure of tremor amplitude. It represents the ratio between the arm’s average path length and the length of a straight line connecting the initial and final positions. The CI indicates movement homogeneity, with higher values indicating the greater amplitude of kinetic tremor.

Repetitive tapping of index finger and thumb, a commonly used task in clinical practice for evaluating bradykinesia (Antonini et al. 2013; Pal and Goetz 2013), was assessed in three 15-second recordings for each side. Linear regression technique was used to determine the movement velocity (expressed in degrees/s).

### Statistical analysis

For clinical data, scores from MDS-UPDRS part III were considered for rest tremor and bradykinesia, whereas scores from FTM-TRS were used for postural and kinetic tremor. For kinematic data, only the values from the tracks that showed a tremor were calculated for rest tremor, while values from all the tracks from the whole sample were considered for action tremor and movement speed. For movement speed, the slowest side was considered in patients for the analysis, whereas the average of the two body sides was considered for HC. The data distribution was assessed visually and using the Shapiro-Wilk test. Categorical variables were presented as frequencies and compared using Fisher’s exact test. Quantitative data were compared using Mann-Whitney U tests. We first compared the data obtained from patients with and without parkinsonian soft signs (ET-plus vs. ET). Then subgroups were formed considering the presence of parkinsonian soft signs in the clinical evaluation and compared using Kruskal–Wallis one-way analysis of variance (ANOVA), with pairwise comparisons. An ANOVA was performed for each parameter. We also performed Spearman’s correlation analyses to investigate potential relationships between clinical and kinematic data of the different couples of symptoms. The data were corrected with False Discovery Rate (FDR) for multiple comparisons. We employed a binomial test to determine if the prevalence of symptoms significantly deviated from a random distribution between the dominant and non-dominant sides. In patients with asymmetry in rest tremor and bradykinesia, we used Fisher’s exact test to evaluate the concordance of the most affected side for both clinical and kinematic data. A similar analysis was performed in the subgroup of patients exhibiting asymmetry in postural and/or kinetic tremors to assess the concordance with the parkinsonian soft signs. All results are presented as mean values ± 1 SD. The level of significance was set at *p* < 0.05. Data analysis was performed using IBM SPSS Statistics for Windows, version 26 (IBM Corp., Armonk, N.Y., USA).

## Results

### Clinical and neurophysiological data and correlates

The sample comprised 35 females (42.68%) and 47 males (57.32%). The mean age ± 1 standard deviation (SD) was 66.93 ± 13.32 years, with an age of onset of 53.66 ± 19.08 years and a disease duration of 13.27 ± 13.92 years. Patients undergoing tremor treatment were evaluated after a 48-hour medication discontinuation. The HC group matched for gender and age was composed by 46 females (57.50%) and 34 males (42,50%), with an age of 65.20 ± 8.84 (all *p* > 0.05).

Among the 82 participants, the ET-plus patients (*n* = 46, 56.10%) and those without parkinsonian soft signs (*n* = 36, 43.90%) had comparable mean ages (ET-plus: 68.33 ± 10.05 years; ET: 65.14 ± 16.58 years, *p* = 0.93). Again, in ET-plus and ET, the ages of onset were 53.80 ± 18.99 and 53.47 ± 19.45 years, respectively (*p* = 0.91). The disease duration was comparable in the two subgroups (ET-plus: 14.52 ± 14.95 years; ET: 11.67 ± 12.52 years, *p* = 0.28). Family history of tremor was observed in 24 (52.17%) ET-plus and 20 (55.56%) ET patients (*p* = 0.76).

**Fig. 1:**
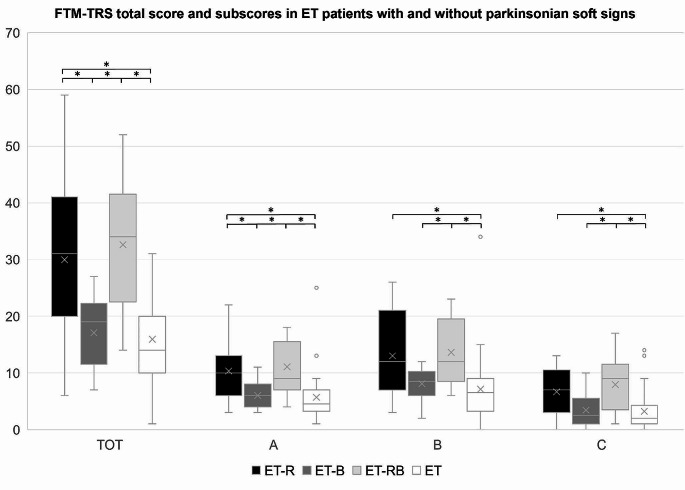
Fahn-Tolosa-Marin Tremor Rating Scale (FTM-TRS) total score and subscores in essential tremor (ET) patients with rest tremor, bradykinesia, both, and with no parkinsonian soft signs. TOT: FTM-TRS total score; A: FTM-TRS subscore A; B: FTM-TRS subscore B; C: FTM-TRS subscore C. For each score, mean (X), median, interquartile range, minimum, and maximum are shown. Asterisks indicate significant comparisons

Among the 46 patients with ET-plus, rest tremor (ET-R) was observed in 19 (41.30%), bradykinesia (ET-B) in 14 (30.43%) and rest tremor plus bradykinesia (ET-RB) in 13 (28.26%) cases. MCI was observed in 24 (52.17%) ET-plus patients and ITG in 9 (19.57%) ET-plus patients, both with no differences in prevalence in subgroups (Table 1). Although there were no statistically significant differences between the demographic data in the subgroups (Table 1, Supplementary material), a trend toward a higher age of onset was observed in ET-RB, and a longer tremor duration in ET-R. Patients with rest tremor (alone or in combination with bradykinesia) showed a higher number of body parts involved in action tremor and higher FTM-TRS scores than patients without rest tremor (Table 1; Fig. 1). In MDS-UPDRS part III, these patients showed higher rest tremor amplitude and constancy (items 3.17 and 3.18). Patients with bradykinesia (alone or in combination with rest tremor) as expected exhibited higher MDS-UPDRS part III scores across all tasks of repetitive movements (items 3.4–3.8). Detailed FTM-TRS and MDS-UPDRS part III scores in the subgroups are shown in the Supplementary material.

**Table 1:** Clinical demographic data of essential tremor (ET) patients with rest tremor, bradykinesia, both, and with no parkinsonian soft signs

	ET-R (*n* = 19)	ET-B (*n* = 14)	ET-RB (*n* = 13)	ET (*n* = 36)	H(3)	*p*
Sex	8 F (42.11%)	4 F (28.57%)	8 F (61.54%)	15 F (41.67%)	-	0.41
Age (years)	67.32 ± 9.44	67.07 ± 13.23	71.15 ± 6.56	65.14 ± 16.58	0.864	0.83
Age of onset (years)	48.79 ± 21.26	53.07 ± 20.06	61.92 ± 11.23	53.47 ± 19.45	3.229	0.36
Tremor duration (years)	18.53 ± 18.70	14.00 ± 12.00	9.23 ± 10.04	11.67 ± 12.52	3.613	0.31
Family history	7Y (36.84%)	10Y (71.43%)	7Y (53.85%)	20Y (55.56%)	**-**	0.27
N° of body segments	2.90 ± 1.37	2.00 ± 1.18	3.46 ± 1.51	1.92 ± 1.20	16.148	**< 0.01** ^**a**^
FTM-TRS total score	29.95 ± 14.92	17.08 ± 6.54	32.62 ± 12.11	15.92 ± 12.01	23.375	**< 0.01**
FTM-TRS section A	10.32 ± 5.07	6.00 ± 2.39	11.08 ± 4.75	5.69 ± 4.21	23.149	**< 0.01**
FTM-TRS section B	13.00 ± 7.20	8.06 ± 2.83	13.62 ± 5.55	7.11 ± 5.84	19.727	**< 0.01**
FTM-TRS section C	6.63 ± 4.50	3.03 ± 3.14	7.92 ± 5.25	3.11 ± 3.41	15.647	**< 0.01**
MDS-UPDRS III	8.53 ± 3.53	8.50 ± 5.76	21.33 ± 8.73	3.87 ± 1.98	49.772	**< 0.01**
MoCA	24.78 ± 2.59	22.86 ± 3.27	23.81 ± 4.35	25.41 ± 2.52	6.835	0.08
MCI	10 (52.56%)	9 (64.29%)	5 (38.46%)	**-**	**-**	0.41
ITG	1 (5.3%)	4 (28.6%)	4 (30.8%)	**-**	**-**	0.12

ET-R: ET-plus patients with rest tremor; ET-B: ET-plus patients with bradykinesia; ET-RB: ET-plus patients with both rest tremor and bradykinesia; ET: ET patients without parkinsonian soft signs; H(3): H statistic (degrees of freedom); F: females; Y: yes; FTM-TRS: Fahn-Tolosa-Marin Tremor Rating Scale; MDS-UPDRS: Movement Disorder Society-sponsored revision of the Unified Parkinson’s Disease Rating Scale; MoCA: Montreal Cognitive Assessment; MCI: mild cognitive impairment; ITG: impaired tandem gait. The subgroup size is indicated in the header. Data are indicated as mean ± standard deviation of the mean. For qualitative variables, percentages are indicated in brackets. False discovery rate for multiple comparisons was applied, and only adjusted p-values are shown. Significant p values in bold. ^a^ ET-R vs. ET *p* = 0.04; ET-B vs. ET-RB *p* = 0.05; ET-RB vs. ET *p* < 0.01. Other pairwise comparisons are reported in the Supplementary material.

Table 2 presents the kinematic characteristics in the four subgroups of ET patients and in HC. Rest tremor was kinematically demonstrated in 14 ET-R patients, 4 ET-B patients, 9 ET-RB patients, and 20 ET patients. In accordance with the clinical data, patients with rest tremor (ET-R and ET-RB) showed higher postural tremor amplitude than ET-B, and the former group also than the ET patients. Notably, the four subgroups had no differences in kinetic and rest tremor amplitude, the latter probably influenced by the low constancy of rest tremor and the smaller size of the subgroups included in this analysis. The tremor frequency was similar between the subgroups for the different conditions (Table 2). As expected, both the whole sample of patients and the different subgroups exhibited slower movement velocity than the HC group. No significant difference was observed between ET subgroups in movement speed, but a role of group size on this finding cannot be excluded.

**Table 2:** Kinematic data of essential tremor (ET) patients with rest tremor, bradykinesia, both and with no parkinsonian soft signs, and in the healthy control (HC) group

	ET-R (*n* = 19)	ET-B (*n* = 14)	ET-RB (*n* = 13)	ET (*n* = 36)	HC (*n* = 80)	H(3)	*p*
PT amplitude	0.28 ± 0.39	0.05 ± 0.02	0.17 ± 0.12	0.17 ± 0.42	NA	15.668	**< 0.01** ^**a**^
PT frequency	6.00 ± 1.27	6.41 ± 1.78	5.66 ± 0.94	6.10 ± 1.33	NA	2.123	0.55
KT amplitude (CI)	1.06 ± 0.03	1.07 ± 0.04	1.06 ± 0.05	1.19 ± 0.80	NA	5.192	0.16
RT amplitude	0.05 ± 0.06	0.02 ± 0.01	0.09 ± 0.12	0.07 ± 0.22	NA	4.388	0.10
RT frequency	6.21 ± 1.60	8.26 ± 2.38	6.16 ± 2.13	6.20 ± 1.56	NA	4.865	0.18
FT velocity	722.48 ± 356.23	797.22 ± 279.03	860.77 ± 244.94	878.46 ± 248.68	1114.12 ± 214.37	39.664	**< 0.01** ^**b**^

ET-R: ET-plus patients with rest tremor; ET-B: ET-plus patients with bradykinesia; ET-RB: ET-plus patients with both rest tremor and bradykinesia; ET: ET patients without parkinsonian soft signs; PT: postural tremor; KT: kinetic tremor; CI: curvature index; RT: rest tremor; FT: finger tapping. For postural and rest tremor, amplitude is expressed in GRMS^2^ and frequency in Hz. Kinetic tremor amplitude is represented by the curvature index. FT velocity is expressed in degrees/s. Each parameter represents the average of the two sides. NA: not applicable. For rest tremor, data refer to tracks presenting tremor. The subgroup size is indicated in the header. Data are indicated as mean ± standard deviation of the mean. False discovery rate for multiple comparisons was applied, and only adjusted p-values are shown. Significant p values in bold.

^**a**^ ET-R vs. ET-B *p* < 0.01; ET-B vs. ET-RB *p* = 0.03; ET-B vs. ET *p* = 0.02. ^**b**^ Patients (whole sample) vs. HC *p* < 0.01; ET-R vs. HC *p* < 0.01; ET-B vs. HC *p* < 0.01; ET-RB vs. HC *p* = 0.02; ET vs. HC *p* = 0.02.

**Fig. 2:**
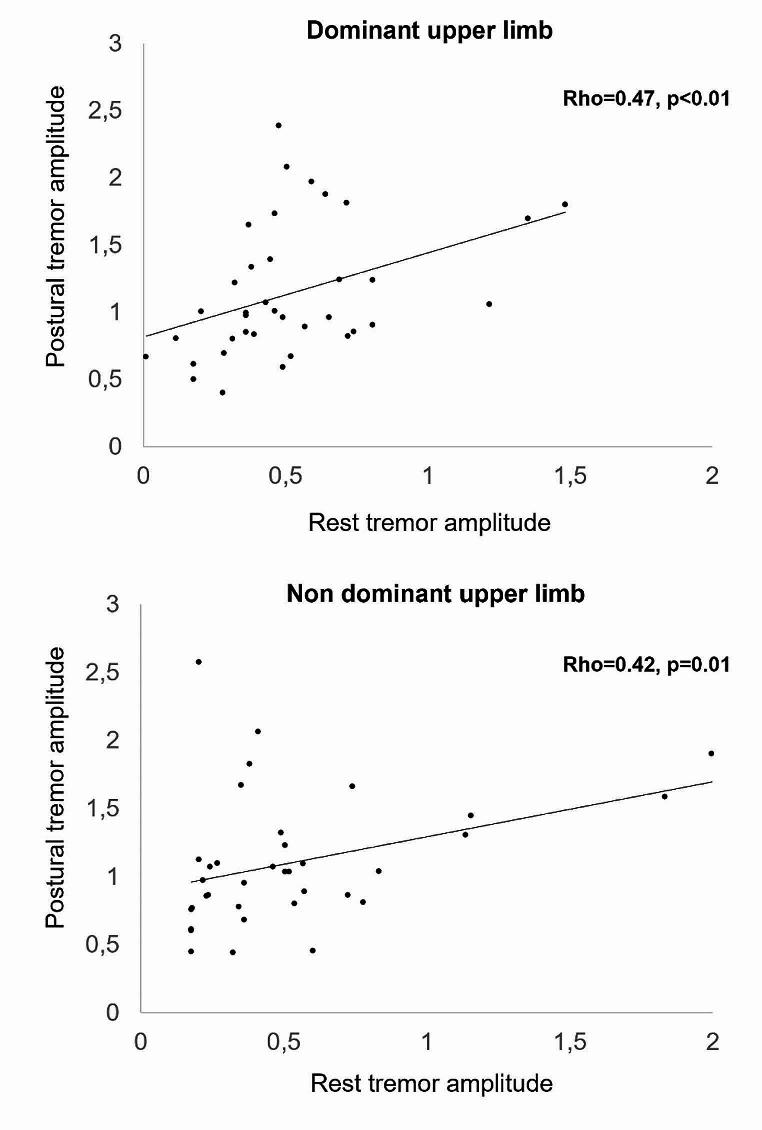
Correlation analysis of kinematic data of rest and postural tremor amplitude in the whole sample of essential tremor (ET) patients. Upper panel: dominant upper limb; lower panel: non dominant upper limb. Tremor amplitude is represented as GRMS^2^. For graphical representation, the original data, spanning a wide range and with small values, underwent a logarithmic transformation using a base-10 scale. To enhance visual clarity and restrict the display to positive values, a constant (i.e., 2) was added to the transformed data

Correlation analysis unveiled a positive relation between the amplitude of rest and postural tremor in both clinical and kinematic assessment (Table 3; Fig. 2). These findings suggest that an higher rest tremor amplitude is associated with a more pronounced postural tremor in the corresponding hand. Conversely, no correlation was observed between the severity of movement velocity during finger tapping and rest and action tremor. No correlations were observed between MoCA scores and clinical and kinematic motor data (Table 3).

**Table 3:** Correlation analysis of clinical and kinematic data of different symptoms in essential tremor (ET) patients

	Clinical data		Kinematic data	
	Rho	*p*	Rho	*p*
Rest tremor – Bradykinesia (FT velocity)				
Dominant arm	0.23	0.04	-0.22	0.18
Non-dominant arm	0.20	0.08	-0.07	0.69
Rest tremor - Postural tremor				
Dominant arm	0.42	**< 0.01**	0.47	**< 0.01**
Non-dominant arm	0.19	0.10	0.42	**0.01**
Rest tremor - Kinetic tremor				
Dominant arm	0.15	0.21	0.35	0.03
Non-dominant arm	0.14	0.24	0.20	0.22
Bradykinesia (FT velocity) - Postural tremor				
Dominant arm	0.03	0.82	-0.24	0.04
Non-dominant arm	0.11	0.35	-0.20	0.11
Bradykinesia (FT velocity) - Kinetic tremor				
Dominant arm	0.18	0.13	-0.07	0.54
Non-dominant arm	0.10	0.40	-0.03	0.78
MoCA - Rest tremor				
Dominant arm	-0.12	0.33	-0.16	0.36
Non-dominant arm	-0.16	0.22	-0.20	0.26
MoCA - Bradykinesia (FT velocity)				
Dominant arm	-0.08	0.51	-0.18	0.15
Non-dominant arm	-0.32	0.01	-0.14	0.26
MoCA - Postural tremor				
Dominant arm	0.11	0.37	0.12	0.37
Non-dominant arm	-0.24	0.05	-0.02	0.90
MoCA – Kinetic tremor				
Dominant arm	-0.07	0.58	-0.03	0.81
Non-dominant arm	0.07	0.55	0.01	0.93

FT: finger tapping; MoCA: Montreal Cognitive Assessment; Rho: Spearman’s correlation coefficient. Significant p values after correction for multiple comparison in bold.

### Asymmetry and side concordance of motor symptoms

Among the patients with rest tremor, 16 out of 32 (50.00%) exhibited asymmetry in the clinical assessment, while for bradykinesia asymmetry was observed in 18 out of 27 (66.67%) cases. Regarding postural and kinetic tremor, asymmetry was present respectively in 38 out of 82 (46.34%) and 29 out of 74 (39.19%) patients. The side with more severe rest tremor, bradykinesia and postural tremor was randomly distributed between dominant and non-dominant sides (all *p* > 0.05) (Supplementary material). The kinetic tremor was observed to be more prominent on the non-dominant side, but further analysis of the kinematic data did not confirm this finding [Dominant: 41/79 (51.90%), Non-dominant: 38/79 (48.10%), *p* = 0.82].

Interestingly, in clinical assessment no significant side concordance was found between rest tremor and bradykinesia, as well as between these motor symptoms and action tremor (Table 4). The analysis on the kinematic data confirmed this finding, showing no difference in the percentages of patients with concordant and non-concordant pairs of symptoms (Table 4).

**Table 4:** Clinical and kinematic data of side concordance of parkinsonian soft signs and action tremor in essential tremor (ET) patients

	Concordant	Non-concordant	*p*
Clinical data			
Rest tremor - Bradykinesia	4/4 (100%)	0/4 (0%)	0.33
Rest tremor - Postural tremor	7/10 (70%)	3/10 (30%)	0.53
Rest tremor - Kinetic tremor	1/4 (25%)	3/4 (75%)	1
Bradykinesia - Postural tremor	6/7 (85.71%)	1/7 (14.29%)	0.14
Bradykinesia - Kinetic tremor	4/7 (57.14%)	3/7 (42.86%)	1
Kinematic data			
Rest tremor - Bradykinesia	21/45 (46.67%)	24/45 (53.33%)	0.76
Rest tremor - Postural tremor	27/44 (61.36%)	17/44 (38.64%)	0.22
Rest tremor - Kinetic tremor	23/42 (54.76%)	19/42 (45.24%)	0.76
Bradykinesia - Postural tremor	42/75 (56.00%)	33/75 (44.00%)	0.36
Bradykinesia - Kinetic tremor	42/78 (53.85%)	36/78 (46.15%)	0.65

The two signs were defined as concordant when they were more severe on the same body side (dominant or non-dominant). Percentages are indicated in brackets.

In summary, data demonstrate that rest tremor and bradykinesia in ET patients, as well as action tremor, are frequently asymmetrical. However, no side concordance is observed in both clinical and kinematic assessment, suggesting that distinct pathophysiological mechanisms may mediate these parkinsonian soft signs.

## Discussion

In this study, we comprehensively examined clinical and kinematic features of rest tremor and bradykinesia in a relatively large sample of ET patients. Our focus was on their relationship with action tremor, as well as on the side distribution, i.e., asymmetry and side concordance, of these symptoms. Correlation analysis revealed a relation between the severity of rest and postural tremor, while no relationship was present between rest or postural tremor and bradykinesia. Although clinical evaluation showed an asymmetric distribution of rest tremor and bradykinesia in a large proportion of patients, no side concordance was observed between the two parkinsonian soft signs in both clinical and kinematic assessments. These results suggest that the two soft signs in ET may originate from distinct pathophysiological mechanisms.

Our results are consistent with the relatively high prevalence of rest tremor and bradykinesia in ET (Bologna et al. 2020; Erro et al. 2022a, b, c), emphasizing their clinical significance. Notably, in our sample bradykinesia was more frequent when compared with other studies with similar populations (Bologna et al. 2020; Erro et al. 2022b) because it was among the inclusion criteria for ET-plus subjects, and also because of the lower diagnostic threshold (i.e., at least 1 point in the relevant MDS-UPDRS item) we choose to identify slight motor abnormalities. Accordingly, the comparison with the HC group confirmed a lower velocity during finger tapping in patients. A similar finding in ET has been clearly demonstrated in previous neurophysiological studies (Paparella et al. 2021b), including studies on repetitive finger movements (Farkas et al. 2006; Jiménez-Jiménez et al. 2010; Costa et al. 2010; Bologna et al. 2020). A novel aspect in the present study is the comprehensive analysis of the clinical correlates of rest tremor and bradykinesia in ET. Our data revealed that patients with rest tremor showed more severe and widespread action tremor. These data may suggest a more severe involvement of the cerebello-thalamo-cortical circuit (van den Berg and Helmich 2021) in this subgroup. This finding is consistent with our recent observation in patients with valproate-induced tremor, where underlying cerebellar dysfunction resulted in a more extensive distribution of tremor throughout the body and a higher occurrence of rest tremor (Paparella et al. 2021a; De Biase et al. 2022). This is also in line with what has been observed as the disease progresses over time, (Louis 2020; Angelini et al. 2023). Consistent with this hypothesis, the correlation analysis demonstrated a positive relationship between the amplitude of rest and postural tremor in kinematic assessment. Common pathophysiological bases, with oscillations in the same cerebral network, may underlie these two symptoms.

In patients with ET and bradykinesia, higher MDS-UPDRS scores were observed in repetitive movement tasks, as expected. However, no differences were found in action tremor compared to other subgroups. Correlation analysis indicated no significant association between bradykinesia and action tremor, suggesting that bradykinesia represents a distinct characteristic of ET. It is indeed present at different degrees in all subgroups compared to healthy subjects, and is independent of other main motor manifestations. These findings suggest the involvement of separate pathophysiological mechanisms for bradykinesia and rest tremor in ET, as it is also considered to occur in PD (Chen et al. 2022). In the present paper, we considered bradykinesia as movement slowness, as it is a common movement alteration in ET patients (Bologna et al. 2020; Paparella et al. 2023). There is evidence, however, that the combination of components of the bradykinesia complex (Bologna et al. 2023) may differ in ET from other movement disorders (Bologna et al. 2020; Paparella et al. 2023). Further studies on the characterization of bradykinesia in ET, and analyses on the role of its different components and their relationship to other motor symptoms are needed.

The cognitive results confirmed the high risk and prevalence of MCI in ET patients (Louis et al. 2019; Bologna et al. 2019; Angelini et al. 2023) without differences in prevalence in ET-plus subgroups with parkinsonian signs. However, correlation analysis allowed us to exclude a direct influence of cognitive disturbances on motor signs in our sample. Similarly, no differences were observed in the prevalence of ITG in the various subgroups. The relationships between voluntary movement alterations and cognitive disturbances in ET need to be further investigated in specifically designed studies. 

Further insights into the pathophysiology of parkinsonian soft signs in ET can be gained from analyzing these signs’ asymmetry and side concordance. Consistent with previous findings (Cohen et al. 2003; Louis et al. 2011, 2015; Delgado et al. 2022), our data provide evidence of asymmetric rest tremor in a high proportion of patients. Bradykinesia was also frequently asymmetric, as well as, to a lesser extent, the action tremor. These findings suggest mechanisms of lateralization similar to those hypothesized in other neurodegenerative diseases (Lubben et al. 2021). Data on the relationship between the asymmetry of action tremor amplitude and handedness are conflicting (Hornabrook and Nagurney 1976; Louis et al. 1998; Machowska-Majchrzak et al. 2011; Biary and Koller 1985; Putzke et al. 2006), and the role of handedness in parkinsonian soft signs in ET has never been addressed. Our results do not show a clear side prevalence of these motor features as some evidence has shown in PD (van der Hoorn et al. 2012). Regarding the side concordance of motor manifestations in ET, only studies on rest and action tremor are available, and some of them showed side concordance of the two motor signs (Louis et al. 2011, 2015; Delgado et al. 2022), but opposite evidence exists (Cohen et al. 2003). In a recent study, Delgado and colleagues demonstrated that concordant pairings of asymmetrical rest tremor and action tremor are more common than discordant (Delgado et al. 2022). Our results do not suggest side concordance between action tremor and parkinsonian soft signs in ET and do not reveal co-occurrence of rest tremor and bradykinesia on the same side, from both a clinical and kinematic perspective. These findings strengthen the hypothesis that different mechanisms with a different distribution in the central nervous system may underly the heterogeneous motor symptoms in ET.

Concerning the possible pathophysiology of parkinsonian soft signs in ET, some neurotransmitter neuroimaging studies (Lee et al. 1999; Ceravolo et al. 2008; de Verdal et al. 2013) showed nigrostriatal dopaminergic denervation in ET patients with rest tremor. Structural and functional neuroimaging studies in these patients also showed an involvement of basal ganglia, mainly globus pallidus (Nicoletti et al. 2015; Caligiuri et al. 2017). In addition, the cerebellum also seems to play a role in ET with rest tremor (Novellino et al. 2016; Prasad et al. 2018), in the context of the involvement of the cerebello-thalamo-cortical circuit in tremor genesis (van den Berg and Helmich 2021). Considering that dopamine influences the cerebellum in the ET (Kosmowska and Wardas 2021), and that the cerebellum itself may modulate dopamine levels in the basal ganglia (Washburn et al. 2024), it is plausible that an abnormality in the interplay between the basal ganglia and cerebellum, potentially mediated by the same neurotransmitter, could account for the rest tremor observed in ET. In relation to bradykinesia in ET, it has been suggested that similar to PD it may arise from a network dysfunction involving the cerebellum, basal ganglia, and sensorimotor cortical areas (Paparella et al. 2021b). Recent evidence also showed that subtle changes in dopaminergic tone in the striatum can play a role in movement slowness in ET (Colella et al. 2023). In ET patients with bradykinesia, the underlying changes may both originate in the cerebellum, disrupting its functions related to velocity coding, or in the basal ganglia component of this network. Differences in the proportion of involvement of these two circuits in ET patients with parkinsonian soft signs may explain the different clinical-neurophysiological correlates in patients with rest tremor and bradykinesia.

Based on our findings, we make some considerations on the concept of ET-plus. The data concerning rest tremor may suggest that it is a feature that manifests in the later stages of the disease, with the tremor intensifying in terms of severity, spreading throughout the body and appearing under different activation conditions (Louis 2020; Angelini et al. 2023). This seems to be confirmed by the trend observed in our data. On the other hand, ET patients with bradykinesia only seem to form a distinct subgroup characterized by less severe tremor and no correlation between bradykinesia and other motor symptoms. The ET-plus category is highly heterogeneous and may encompass different subgroups that display varying relationships to ET, representing distinct entities. These two parkinsonian soft signs should be considered separately in this contest as they appear to have different significance in indicating the state of disease severity, and it is necessary in view of a precise phenotypic characterization of ET manifestations.

It can be argued that patients with both rest tremor and bradykinesia may fall into the diagnostic category of parkinsonism, also in view of the trend of older age in patients with both soft signs that may suggest conversion to a different clinical picture. However, as these signs were of a questionable nature, mild in intensity, and only the speed component of movement was taken into account, there was insufficient evidence to formulate alternative diagnoses. Furthermore, the patients included in this study have been followed in the outpatient clinic for an extended period, and we have confidence in the ET diagnosis, also because of the lack of other typical signs of PD and the slow evolution of symptoms over time. Only in cases where a diagnostic doubt might persist (n. 10), a DAT scan was performed, and all results showed no significant nigrostriatal dopaminergic dysfunction, supporting the absence of alternative conditions. It cannot be ruled out that ET patients with parkinsonian signs represent a subgroup that will develop PD, due to the occurrence of ongoing processes that will manifest over time. Currently, the relationship between ET and PD is debated (Algarni and Fasano 2018; Tarakad and Jankovic 2019). However, this being a cross-sectional study, it cannot provide information on the evolution of symptoms over time. Longitudinal studies are necessary to clarify the relationship between ET and PD.

It is important to acknowledge the possible confounding and limitations of this study. All patients were right-handed, and further studies are needed to explore the relationship between soft signs asymmetry and handedness. Furthermore, we focused here on rest tremor and questionable bradykinesia in ET, whereas the characterization of other mild motor signs was beyond the scope of the study. Some of these motor symptoms, such as questionable dystonia, although minimal and of unclear significance (Becktepe et al. 2021; Pandey et al. 2020) could still have some influence on voluntary movement, so it is necessary for these to be deepened with targeted studies. In addition, only the upper limbs were assessed as they most frequently show the symptoms investigated. The low level of severity we have considered for bradykinesia may include alterations also observed in healthy subjects (Williams et al. 2023; Paparella et al. 2023), and further studies on the role of factors such as ageing in the healthy population will be helpful in clarifying this aspect. Finally, the sample size, though not small, may have some impact on the results, particularly when analyzing smaller subgroups, and larger-scale studies are needed to strengthen our findings.

Our study has some important strengths. First of all, a comprehensive analysis and detailed phenotyping were conducted studying parkinsonian soft signs together with action tremor under different activation conditions. In addition, the clinical data were supported by an objective neurophysiological measurement of tremor and movement speed, which takes into account small alterations invisible to clinical assessment.

In conclusion, by characterizing the parkinsonian soft signs in ET we uncovered clues about the underlying pathophysiology of these symptoms. Our findings suggest that rest tremor and bradykinesia are likely mediated by distinct mechanisms, and contribute differently to the heterogeneity of ET-plus. Future studies should better characterize parkinsonian soft signs in ET to unravel their clinical significance and their pathophysiology.

### Supplementary Material

1. Pairwise comparisons of demographic data shown in Table 1.

2. Supplementary Table 1: Fahn-Tolosa-Marin Tremor Rating Scale (FTM-TRS) detailed scores in essential tremor (ET) patients with rest tremor, bradykinesia, both of them and with no parkinsonian soft signs.

3. Supplementary Table 2: Movement Disorder Society-sponsored revision of the Unified Parkinson’s Disease Rating Scale (UPDRS) part III detailed scores in essential tremor (ET) patients with rest tremor, bradykinesia, both of them, and no parkinsonian soft signs.

4. Supplementary Table 3: Clinical data of asymmetry and side prevalence of parkinsonian soft signs and action tremors in essential tremor (ET) patients.

## Data Availability

The data supporting the findings of this study are available on request from the corresponding author. The data are not publicly available due to privacy or ethical restrictions.

## Abbreviations

ANOVA: analysis of variance
CI: curvature index
DAT: dopamine transporter
ET: essential tremor
ET-B: ET-plus patients with bradykinesia
ET-plus: essential tremor-plus
ET-R: ET-plus patients with rest tremor
ET-RB: ET-plus patients with rest tremor and bradykinesia
F: females
FDR: False Discovery Rate
FT: finger tapping
FTM-TRS: Fahn-Tolosa-Marin Tremor Rating Scale
GRMS: acceleration root mean square
HC: healthy control
ITG: impaired tandem gait
MCI: Mild cognitive impairment
MDS-UPDRS: Movement Disorder Society-sponsored revision of the Unified Parkinson’s Disease Rating Scale
MoCA: Montreal Cognitive Assessment
P1: posture 1
P2: posture 2
PD: Parkinson’s disease
Rho: Spearman’s correlation coefficient
RMS: root man square
SARA: Scale for the Assessment and Rating of Ataxia
SD: standard deviation
SPECT: single-photon emission computed tomography
Y: yes
